# Self-efficacy, mindfulness, and perceived social support as resources to maintain the mental health of students in Switzerland’s universities of applied sciences: a cross-sectional study

**DOI:** 10.1186/s12889-024-17692-x

**Published:** 2024-01-31

**Authors:** Myriam Guzman Villegas-Frei, Jonathan Jubin, Claudia Ortoleva Bucher, Annie Oulevey Bachmann

**Affiliations:** La Source School of Nursing, HES-SO University of Applied Sciences and Arts Western Switzerland, Av. Vinet 30, Lausanne, 1004 Switzerland

**Keywords:** Resources, Students of applied sciences, Mental health, Protective factors, Self-efficacy, Perceived stress, Social support, Mindfulness

## Abstract

**Background:**

Switzerland’s student population is at a particularly high risk of developing mental health disorders, creating a major challenge for Switzerland’s higher education establishments. Research to date has primarily sought to identify the risk factors affecting students’ mental health; however, their exposure to these factors is often unavoidable. Thus, the present study adopted a salutogenic approach focussing on the determinants of health. We examined the mental health resources available to students reported in the literature as being susceptible to helping them maintain good mental health despite their exposure to risk factors.

**Methods:**

In February 2020, 2,415 first- and second-year bachelor’s degree students in applied sciences in French-speaking Switzerland completed an online questionnaire. The variables measured were self-evaluated mental health, perceived stress and three potential health resources: students’ feelings of self-efficacy, their capacity for mindfulness and their social support. The results were analysed using hierarchical linear regression models.

**Results:**

When all the variables were included in the model without interaction effect, our results revealed that students’ self-evaluated mental health was negatively associated with perceived stress (β = -0.43, *p* < 0.001) and positively associated with the three potential health resources (self-efficacy: β = 0.26, *p* < 0.001; mindfulness: β = 0.10, *p* < 0.001; social support: β = 0.17, *p* < 0.001). An analysis of the interaction effects also revealed that a high level of self-efficacy was associated with perceived stress being less strongly linked to mental health (β = 0.29, *p* < 0.001).

**Conclusions:**

These findings suggest that self-efficacy, mindfulness, and perceived social support are valuable resources for protecting students’ mental health. Thus, implementing interventions aimed at reinforcing them, could support students in applied sciences all along their academic pathway, in their classes and during their professional work experience placements.

## Background

According to the World Health Organization, good mental health is a state of psychological well-being that forms an integral part of an individual’s capacity to live a fulfilling life; that is, to be able to develop and maintain personal relationships, study, work or enjoy leisure activities or make day-to-day decisions about one’s education, work, home chores and other issues [1]. Self-perceived mental health is a subjective measure of overall mental health. It does not equate to any validated measures of mental health disorders but is an individual’s perception of their personal state of mental health [2–4].

Young adults are generally perceived as being in naturally good health [5–7]. However, epidemiological studies have shown that this population is living through a particularly delicate period in life, particularly with regard to mental health [8–10]. Indeed, half of mental health disorders present before around 21 years of age and half of severe psychiatric disorders appear before 26 [11]. Worldwide and in Switzerland, suicide is the second most frequent cause of death among young people [12, 13]. In Switzerland, the proportion of young people aged between 16 and 25 presenting with moderate to severe symptoms of depression increased from 10.4% in 2012 to 13.5% in 2017 [7]. A recent representative study in Switzerland showed that a quarter of young adults screened positive for common mental health disorders, indicating their rising prevalence [14]. Furthermore, the current generation of young adults presents a greater risk of developing mental disorders than previous ones [11]. A mix of biological, psychological and social factors help to explain this age group’s worsening mental health over the last few decades [7, 15–17]. The COVID-19 pandemic accelerated this phenomenon due to the general anxiety it generated, as did lockdown measures and their associated hardships [18–25].

The situation is even more worrying among undergraduate students [26–29]. They present with a greater risk of developing mental health or other behavioural disorders than their peers in the same age group in the general population [27, 30]. Indeed, they display a high level of self-perceived stress [31–33], and between 2013 and 2021, their levels of mental distress increased (depression, anxiety, non-suicidal self-mutilation, eating disorders) and their levels of fulfilment diminished [34]. The most dangerous period seems to be the first semester of a student’s first year, possibly because it is such a critical transition period [27]. Indeed, it is a major transition period in their social, family and personal lives [23, 23] In Switzerland, 18% of students stated that they had a long-term general health disorder, and among these, 26% mentioned that psychological troubles were the disorders that most impeded their education [35].

This situation can be partly explained by undergraduate students’ exposure to numerous different stressors that can have a negative influence on their mental health [36]. These can include: their expectations of themselves and others’ expectations of them; new types of social relationships; a new living environment; the higher standards required in tertiary education; financial difficulties; a lack of time; and examinations [36–39]. The link between perceived stress and mental health or psychological well-being is frequently negative [40–42].

The studies mentioned above examined university students’ mental health and the risk factors they are exposed to. The students in Switzerland’s universities of applied sciences have very different profiles, however. Many of them work part-time, at up to 40%, in parallel to their studies, and they carry out professional work experience internships that are graded and are an integral part of their academic training [43]. It is important to examine this population separately because their specific characteristics could expose their mental health to risk factors in a different way to the university students in Switzerland’s ‘classic’ universities.

International data on students’ mental health often focus on identifying risk factors (pathogenesis) such as addictions or dangerous behaviours [44–48] but not the factors protective of their mental health. However, exposure to stressors cannot always be avoided. For this reason, the field of primary prevention and health promotion focuses on the protective factors of health. This approach seeks to develop individuals’ capacities to cope with the stressors they are commonly exposed to in order to ensure that they can maintain their own health status [49]. To do this, we must: (i) identify the factors protective of students’ health, sometimes also called *general health resources* [50]; (ii) develop interventions to teach individuals how to harness these protective factors; and (iii) implement those interventions and evaluate them. This type of salutogenic approach [51] complements primary prevention approaches that seek to limit exposure to risk factors affecting health.

Since the 1970s, the nursing sciences have developed a broad range of care frameworks that have modelled health maintenance and conceptualised the notion of health resources. The Neuman Systems Model is one of these [49]. Like Betty Neuman, the researchers and clinicians who call for a more salutogenic approach to nursing use the following assumptions: (i) a priori, stressors are neither positive nor negative; (ii) individuals have a number of endogenous and exogenous (interpersonal and extra-personal) factors protective of their health at their disposal; (iii) it is necessary to act along both axes of primary prevention by reducing exposure to stressors and harnessing the factors susceptible to protect health; (iv) a factor can be considered protective if it moderates the relationship between the predictor (the explanatory variable) and the health outcome (the dependent variable) [52].

In most of the papers examined when preparing the present study, the terms ‘stress factor’ and ‘stressor’ were used without differentiating between them, implying, de facto, that both negatively affect health. However, as we saw above, stressors can, a priori, be thought of as neutral: their impact on health can vary depending on the circumstances and the moment. Exposure to a stressor can, indeed, manifest itself as stress [27, 53] and affect students’ mental health [54–56], but it can also be eustress that does not negatively affect their mental health because it is perceived as being positive [49]. Occurrences and manifestations of stressors’ effects depend on individuals’ perceptions of them and capacities to cope with them [49]. In the present study, perceived stress was used as a proxy to estimate exposure to stressors. Indeed, objectively measuring exposure is not possible, as it would require a list of all stressors to which students might be exposed, which does not exist as this would be highly dependent on the context in which students live and study.

To identify the factors that enable individuals to deal with these stressors without negatively affecting one’s health—which are, therefore, potentially protective of perceived mental health—we performed an integrative literature review of this topic. The three factors which seemed the most likely to protect students’ mental health were feelings of self-efficacy, a capacity for mindfulness and social support.

Feelings of self-efficacy correspond to a person’s feelings about their capacities to harness their self-motivation and cognitive resources and then adopt the necessary behaviours to take full control of the tasks they have to complete or to deal with the circumstances that they encounter [57]. Strong feelings of self-efficacy improve personal performance and well-being; they facilitate people trying to accomplish something or complete activities, and they reduce vulnerability to depression.

The faculty of mindfulness is defined as a “state of being attentive to and conscious of what is happening in the present” [58]. Among adults, a strong capacity for mindfulness has been associated with well-being and lower scores for depression, anxiety and stress [58].

Perceived social support is the assistance and support that an individual perceives they receive from others or their perception of the general readiness of their entourage, such as family and friends, to provide them with the psychological and material resources they need [59]. Social support enables individuals to adjust their lives to stressors [60].

These potential factors protective of health could thus become the targets of specific primary prevention and mental health promotion interventions for young adults, particularly among the undergraduate students in Switzerland’s universities of applied sciences. Indeed, acting preventively in favour of this population’s mental health reduces occurrences of negative mental health effects and, in some cases, helps to avoid them [61, 62]. There has been, however, a lack of epidemiological data from Switzerland to legitimise the implementation and funding of such interventions [7], particularly with regard to undergraduate students [63]. Likewise, there are very few data on the mental health of students in Switzerland’s universities of applied sciences, and there is no knowledge about whether the factors identified above have the potential to help protect their mental health.

Thus, the present study had two primary objectives:

1. Examine the nature of the association between the levels of exposure to stressors perceived by bachelor’s degree students in applied sciences and their mental health.
2. Examine whether feelings of self-efficacy, the capacity for mindfulness and perceived social support are associated with the mental health of first- and second-year bachelor’s degree students in applied sciences and whether these factors moderate the relations between mental health and perceived levels of exposure to stressors.

## Methods

### Design and participants

We selected a descriptive correlational study design to attain our objectives. Our available convenience sample study population during the 2017–2018 academic year was the 11,500 first- and second-year students at the University of Applied Sciences and Arts Western Switzerland (HES-SO) [64, 65]. The inclusion criteria were being an officially enrolled first- or second-year bachelor’s degree student in a university of applied sciences and being able to read and understand French or English.

### Procedure

This study sought to examine students from the 26 specialised colleges comprising the University of Applied Sciences and Arts Western Switzerland (HES-SO). These are spread out over seven cantons (Jura, Bern, Neuchâtel, Geneva, Fribourg, Valais and Vaud), and the HES-SO primarily trains students in the fields of Design and the Visual Arts, Economics and Business, Engineering and Architecture, Music and the Performing Arts, Health, and Social Work.

Written authorisation to contact the students at each of the HES-SO’s 26 specialised colleges was sought from their respective management offices, and 24 gave that authorisation and put the research team in contact with an administrator who would be responsible for distributing general information about the study. A standardised information kit, including a video, slides, posters and flyers, was given to each administrator. They were also responsible for transferring two project emails. The first announced the study’s imminent commencement in the college. The second, sent out 2 weeks later, contained an invitation to complete the questionnaire, a detailed information sheet comprising a written informed consent form for participating in the study and a hypertext link to the self-administered online questionnaire that had been prepared using Sphinx IQ2 software. The questionnaire could be completed using a computer, tablet or smartphone, and the total time necessary was about 15 min. On the questionnaire’s first page, participants were asked to click in the box indicating that they consented to participate in our study under the conditions described in the information sheet. If they refused to do this, they could not continue.

Data collection lasted from mid-February to mid-March 2020, just before the outbreak of the COVID-19 pandemic’s first wave in Switzerland. Two reminders were sent out at 1-week intervals. To boost participation rates, the second-to-last question before the questionnaire asked students whether they wanted to participate in a prize draw for a CHF 50 voucher valid in a local supermarket. If they did, the final box asked them to enter their email address. A data manager independent of the research team was tasked with drawing the winners and sending their vouchers.

### Questionnaire

The online questionnaire was pilot tested on five persons with characteristics similar to those of the available sample population. The questionnaires below were included in the online questionnaire. All of them were validated and used with their respective authors’ permission when required.

#### Score for the six items of domain 2, Psychological Quality of Life, from the short World Health Organization Quality of Life scale, WHOQOL-BREF, 26 items [66, 67]

Internal consistency analyses, correlations, discriminatory validity and construct validity using confirmatory factor analysis have all shown that the WHOQOL-BREF has good to excellent psychometric properties for its reliability. It also displays good results in validity testing [66]. The Psychological Quality of Life domain’s test–retest validity after 4 weeks is 0.79. The internal consistency coefficient for this domain ranges from 0.75 to 0.81 [68]. The WHOQOL-BREF is, therefore, a reliable, valid scale, particularly in its psychological dimensions [66]. Two of this scale’s strengths are its versatility and its sub-5-min completion time. Reference standards exist for 23 countries by sex and age [68].

#### Perceived Stress Scale, PSS, 14 items [69]

This scale’s internal consistency has been validated with Cronbach’s α from 0.84 to 0.86 [69]. Its concurrent and predictive validities are supported by its relationship with participants’ numbers of life events and their impacts [69]. This 14-item version was translated into French, and its reliability was evaluated to have a Cronbach’s α of 0.74. There is no consensus as to the scale’s one-dimensional or two-dimensional construction [70, 71]. The scale’s 14 items have demonstrated stability over a period of a year [72].

#### General Self-Efficacy Scale, GSES, 10 items [73, 74]

This scale’s internal consistency has been evaluated as satisfactory, with Cronbach’s α coefficients ranging from 0.76 to 0.90, with most values above 0.80. The criterion validity is supported by positive associations with optimism and satisfaction with one’s work. The scale’s French version was validated in a population of students in France, with good internal consistency (Cronbach’s α > 0.85) [75]. It has also demonstrated stability over time (7 weeks) [76].

#### Mindful Attention Awareness Scale, MAAS, 15 items [58]

This scale’s internal consistency has been validated in numerous samples, including one in a student population (Cronbach’s α from 0.80 to 0.87). Its stability over time was demonstrated by its intra-class correlation of 0.81 over a period of 4 weeks (equivalent to a Pearson’s *r* correlation coefficient) in a population with a mean age of 19 years old [58]. The scale’s French version has also been validated, with Cronbach’s α = 0.84 [77].

#### Multidimensional Scale of Perceived Social Support, MSPSS, 12 items [78]

This scale was developed using a population of 275 university students in the USA. Cronbach’s α ranged from 0.81 to 0.90 for the ‘family’ subscale, from 0.90 to 0.94 for the ‘friends’ subscale, from 0.83 to 0.98 for the ‘significant others’ subscale and from 0.84 to 0.92 for the full MSPSS score [78]. Test–retest reliability ranged from 0.72 to 0.85 over a period of 4 months [78]. The reliability of the French version of the scale was tested and confirmed among a sample of young mothers in France: internal consistency for the full questionnaire displayed a Cronbach’s α = 0.92, with subscale internal consistencies ranging from 0.91 to 0.94 [79].

#### Demographic questionnaire

Participants were also asked to provide demographic information, including gender (woman, man or defines as other), age, living arrangements (with parents, with a partner, in shared accommodation, alone, other), year of studies (first or second year of bachelor’s degree) and whether they worked a part-time job in parallel their studies (yes, no).

### Statistical analyses

We began by performing a descriptive statistical analysis of the collected questionnaire data. We then used a hierarchical linear regression model to determine how the different variables measured were associated with the mental health scores calculated. We entered the variables into the regression model in four separate blocks: (1) sociodemographic variables; (2) perceived stress; (3) factors protective of health (feelings of self-efficacy, capacity for mindfulness and perceived social support); (4) the moderating effects of the three factors protective of health on perceived stress’s effects on mental health (the interaction effects). Categorical variables were included in these analyses as either dichotomised variables or dummy variables. All the statistical analyses were performed using R 4.1.1 software.

## Results

### Descriptive results

A total of 2,415 participants completed the questionnaire in French (2,376) or English (39). The responses of 137 participants were excluded because they stated that they were not first- or second-year bachelor’s degree students at the time they responded to the questionnaire. Two participants withdrew their consent and were excluded from our analyses, leaving 2,276 valid questionnaires.

### Sociodemographic variables

Participants’ sociodemographic characteristics and mean scores are summarised in Tables 1 and 2. The sample comprised 68.4% women, 29.8% of men and 1.8% of participants preferred to describe themselves otherwise. Mean participant age was 22.8 years old (SD = 3.9) and 57.6% were in the first year of their bachelor’s degree. The majority of them lived with their parents (54.4%), and a little less than half were working a part-time job in parallel with their studies (47.2%).

**Table 1:** Descriptive statistics for sociodemographic variables

Variable	N
Gender	
Woman	1556 (68.4%)
Man	678 (29.8%)
Describes as other	42 (1.8%)
Year of study	
First	1305 (57.3%)
Second	971 (42.7%)
Works a part-time job	
Yes	1074 (47.2%)
No	1202 (52.8%)
Living arrangements	
With parents	1238 (54.4%)
With a partner	250 (11.0%)
In shared accommodation	426 (18.7%)
Alone	267 (11.7%)
Other	95 (4.2%)

**Table 2:** Descriptive statistics for numerical variables

	Mean	SD	Min	Max	Cronbach’s α
Perceived mental health (0–100)	63.73	17.39	0	100	0.82
Perceived stress (1–5)	2.96	0.61	1.14	4.79	0.89
Feelings of self-efficacy (1–4)	3.04	0.51	1	4	0.87
Capacity for mindfulness (1–6)	3.84	0.78	1.33	6.0	0.85
Perceived social support (1–7)	5.77	1.06	1	7	0.91
Age	22.8	3.9	17	53	

### Outcome and independent variables

Table 2 presents the distributions of each scale’s scores. The internal consistencies of the numerical scales were calculated using Cronbach’s α coefficient [80].

### Hierarchical regression

In order to determine which variables most affected perceived mental health, we used hierarchical linear regression that included our independent variables and was added to the model in blocks. Associations between these variables and mental health are presented in Table 3.

**Table 3:** Hierarchical linear regression model for mental health

	*Step 1* β	*Step 2* β	*Step 3* β	*Step 4* β
Gender: Man	0.09***	-0.05**	-0.01	-0.01
Gender: Other	-0.03	-0.04*	-0.03	-0.03
Age	0.02	0.03	0.03	0.03
Year of study: Second	0.03	-0.03	-0.02	-0.01
Part-time job: Yes	0.07**	0.04**	0.03*	0.04**
Living arrangement: With a partner	0.00	0.03*	0.01	0.01
Living arrangement: Shared accommodation	-0.03	-0.02	-0.02	-0.02
Living arrangement: Alone	-0.07**	-0.03*	-0.03*	-0.03
Living arrangement: Other	0.02	0.03	0.02	0.02
Perceived stress		-0.71***	-0.43***	-0.94***
Self-efficacy			0.26***	-0.05
Mindfulness			0.10***	0.08
Perceived Social support			0.17***	0.05
Perceived stress * Self-efficacy				0.29***
Perceived stress * Mindfulness				0.02
Perceived stress * Perceived Social support				0.15
Raw *R*^2^	0.021	0.499	0.584	0.591
Adjusted *R*^2^	0.017	0.497	0.582	0.588

Dichotomised and dummy variables: Gender *vs* Woman; Year of Study *vs* First; Part-time job *vs* No; Living arrangements *vs* With parents

*β* Standardized regression coefficient

^*^p < 0.05; **p < 0.01; ***p < 0.001

#### Step 1 – control variables

First, we controlled for associations between gender, age, year of study, working a part-time job, living arrangements and mental health. The analysis revealed that being a man was associated with a better perceived mental health than being a woman (β = 0.09, *p* < 0.001). Furthermore, participants living alone presented with significantly lower mental health scores than those living with their parents (β = -0.07, *p* = 0.001). In contrast, having a part-time job was associated with better perceived mental health than not having one (β = 0.07, *p* = 0.002). Although it was significantly better than an intercept-only model, this model could only explain 2.1% of the variance in the dependent variable (Δ*R*^2^ = 0.021, *F*(9, 2266) = 13.13, *p* < 0.001).

#### Step 2 – perceived stress

By adding participants’ perceived stress to the model, we found that this variable was strongly negatively associated with perceived mental health (β = -0.71, *p* < 0.001). This step also revealed that men (β = -0.05, *p* = 0.002) and participants who described their gender as ‘other’ (β = -0.04, *p* = 0.011) showed a worse mental health than women, which is the opposite as what appeared in step 1. These associations resulting from integrating perceived stress into the model suggest that different genders perceive different levels of stress. The variance explained by the model increased significantly in step 2, to 49.9% (Δ*R*^2^ = 0.478, *F*(1, 2265) = 2367, *p* < 0.001).

#### Step 3 – health resources

Adding health resources to our model revealed that they were all significantly positively associated with perceived mental health (self-efficacy: β = 0.26, *p* < 0.001; mindfulness: β = 0.10, *p* < 0.001; perceived social support: β = 0.17, *p* < 0.001). Except for having a part-time job (β = 0.03, *p* = 0.012) and living alone (β = -0.03, *p* = 0.036), the associations observed between the control variables and perceived mental health in earlier steps were no longer significant after adding this block to the model. This suggests that these associations were at least partly explained by differences linked to these three protective factors. Step 3 also increased significantly the part of the variance in perceived mental health explained by the model to 58.4% (Δ*R*^2^ = 0.085, *F*(3, 2262) = 157.5, *p* < 0.001).

#### Step 4 - moderation

**Fig. 1:**
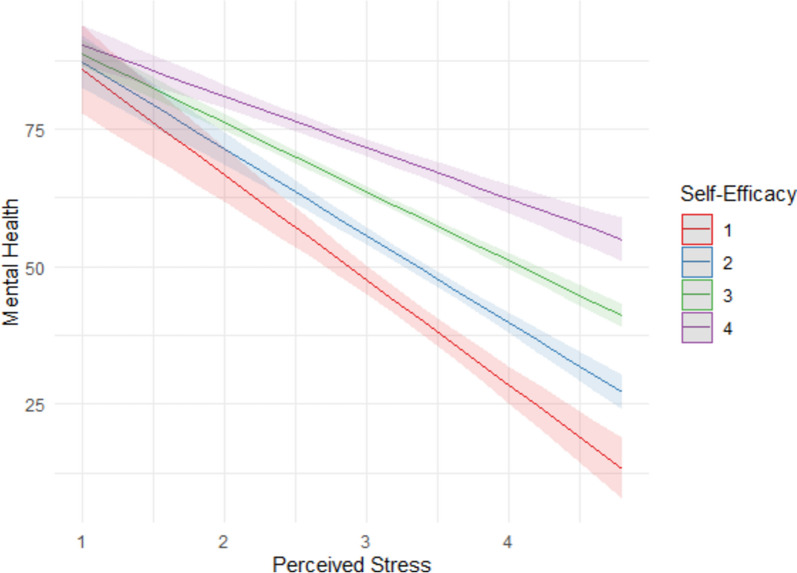
Interaction between perceived stress and self-efficacy scores. The shaded bands represent 95% confidence intervals

Finally, on adding the interactions between the different health resources and perceived stress to the model, the only significant associations with perceived mental health were the level of perceived stress (β = -0.94, *p* < 0.001), having a part-time job (β = 0.04, *p* = 0.008) and the interaction between the perceived stress and self-efficacy (β = 0.29, *p* < 0.001). Adding the interaction effects to the model revealed that the negative association between the perceived stress and perceived mental health was mitigated when levels of self-efficacy were high (Fig. 1). The disappearance of other significant associations is probably due to the fact that the effects of health resources are spread out in the coefficients for the resources themselves and in their respective interaction coefficients (the variable’s first-order effect and the interaction with perceived stress). The increase in the variance of perceived mental health explained by step 4 of the model was only 0.7%. The contribution to the model of the moderating effects of the level of perceived stress was thus significant but weak (Δ*R*^2^ = 0.007, *F*(3, 2259) = 12.8, *p* < 0.001).

## Discussion

The present study’s objective was to identify the health resources that might enable students to protect their mental health when they are exposed to stressors. With reference to the Neuman Systems Model [49], we looked at three factors potentially protective of students’ mental health: their feelings of self-efficacy, their capacity for mindfulness and their perceived social support. We analysed the links between these resources and their perceived mental health, as well as the moderating role that these resources might have on the links between perceived stress and perceived mental health.

Our analyses revealed the existence of a negative association between these two variables. Furthermore, all three health resources identified were positively associated with perceived mental health when controlling for age, gender, year of study, living arrangements and having a part-time job. Including our three potential health resources’ moderating effects on the association between the level of perceived stress and perceived mental health only weakly improved the model’s predictive qualities. Only feeling of self-efficacy significantly moderated the association between perceived stress and perceived mental health. In light of our reference framework—the Neuman Systems Model—feelings of self-efficacy can be considered a health resource for students at the universities of applied sciences in Switzerland.

The association between mental health and self-efficacy in students has already been highlighted. For instance, a study of 3,556 undergraduate and postgraduate students in America revealed a strong correlation between a student’s level of perceived self-efficacy, their academic satisfaction, their motivation and their perceived expectations of faculty [29]. In Europe, a study of 735 students in Norway showed that those reporting suffering from severe mental disorders were four times more likely to report weak feelings of self-efficacy [81].

Bandura described four sources of information favouring feelings of self-efficacy: (i) past performance or success (mastery experiences); (ii) examples to follow (vicarious experiences); (iii) verbal persuasion; and (iv) psychological feedback (affective and somatic states) [57]. The interventions performed to reinforce students’ feelings of self-efficacy, as described in the literature, have been based on these four elements. For mastery experiences, for example, it can be beneficial to offer students workshops or classes in which they can practice their skills before executing them in a real-world professional setting, or to encourage them to actively recall past successful experiences, such as passing exams. The literature describes numerous other strategies, for example, Warner and French [82]. Regarding vicarious experiences, learning by observing peers successfully executing expected behaviours has been recommended. Other pedagogical techniques can also be employed [82]. As to verbal persuasion, lecturers or significant others in a student’s entourage should try to express their belief in the student’s capacities to successfully execute tasks or take on fresh challenges [83, 84]. Students can also develop their abilities to talk about themselves in motivating ways or verbally give themselves advice or use mantras [82]. Finally, it is important that students learn to master or reinterpret their physical or emotional feelings when faced with a difficult task. For example, this could be done by understanding how psychological processes influence biological functions or by learning relaxation or stress management techniques [82].

One meta-analysis involving nursing students noted how a wide range of different interventions aimed at developing their capacities for mindfulness had been able to lower their levels of depression and stress [85]. Another meta-analysis involving medical students noted that training them to develop their capacities for mindfulness contributed to improving their mental health and psychological well-being [86]. The effects on psychological health noted among nursing and medical students can be found among university students in general [87]. Different methods of teaching this capacity to students have been described, including yoga, Mindfulness-Based Stress Reduction (MBSR), Mindful-Based Cognitive Therapy (MBCT), Acceptance and Commitment Therapy (ACT), Dialectical Behaviour Therapy (DBT) and so on. The frequencies of these training sessions varied from 1 h/week to 5 h/week for between 1 and 10 weeks [85]. The effects reported in these studies on developing mindfulness should be treated with care, however, because Dawson and colleagues noted that their quality needed to be improved [87]. A Cochrane review conducted on medical students in 2021 [88] highlighted the importance of, firstly, increasing the number of students involved in studies on the effects of improved capacities for mindfulness and, secondly, gathering long-term data to reduce the risks of bias. Developing students’ capacities for mindfulness remains an interesting avenue towards better mental health and has been shown to increase feelings of self-efficacy among psychology students [89]. Allowing students to develop this capacity could thus also help them to maintain their mental health, but in an indirect manner.

The present study revealed that the perception of receiving strong social support from one’s entourage was positively associated with their perceived mental health. This positive association is consistent with the literature as this association was also found in a 2022 study involving 3,600 students in California spread over ten campuses [90]. Furthermore, a meta-analysis among diverse students populations from all around the world highlighted that high percieved social support is generally associated with low student burnout scores, emphasizing that support is an important part of students’ mental health in many different social and cultural contexts [91].

Encouraging social support from students’ entourages does not, a priori, seem to be the role of educational institutions. Yet, in 2014, the Health Promotion Foundation of Switzerland (HPFS) proposed several personal-level and environmental-level means of reinforcing social support [92]. At the personal level, this could involve reinforcing life skills such as one’s relational capacities, empathy or communication skills. These skills could be trained in some courses or professional internships during studies at universities of applied sciences. At the collective or environmental level, HPFS’s example in school settings could be transferred to tertiary educational settings. Indeed, social support could be made more robust by encouraging peer solidarity, the feeling of truly being a part of a tertiary educational institution, favourable learning environments and student empowerment [92].

### Strengths and limitations

The present study’s principal strength is that it examined a large sample of students at an applied sciences university studying in a wide range of domains. They learn within a pedagogical context quite different from a ‘classic’ university education, as a large part of their practical training occurs during internships in professional settings, and they have thus rarely been studied. In addition, our decision to adopt a salutogenic approach means that our research findings enrich and complete the existing literature on such themes. That literature has generally sought to identify risk factors rather than health resources with the potential to be protective of students’ mental health. Following this approach, it becomes possible to reinforce the resources protective of health, even in situations where exposure to stressors is simply inevitable (as it can be during studies in applied sciences universities).

One important limitation of the study is that we measured undergraduate bachelor’s degree students’ perceived stress using the PSS-14 questionnaire as the closest measurable proxy for the concept of stressors as described in the Neuman Systems Model. Indeed, this scale measures levels of perceived stress—a proxy measurement of true exposure to stressors. It does not objectively measure students’ exposure to stressors. In addition to variance between individuals, the sum effect and nature of these stressors can vary from one domain of study or type of sample group to another. Apart from the stressors commonly described as affecting undergraduate students (e.g. examination, a new academic culture, living away from home, a lack of financial resources), the students in Switzerland’s universities of applied sciences are exposed to the particular stressors of their domain of studies. For example, nursing students doing their internships are exposed to their patients’ suffering and sometimes traumatic deaths. In the performing arts, such as music and theatre, students have to perform in front of live public and a critical audiences. Attempting to draw up an exhaustive list of potential stressors for our student population would have carried the risk that many of those stressors would not apply to all the study participants. Crandall et al. encountered similar difficulties when they attempted to solve the same problem by developing and using the Undergraduate Stress Questionnaire [93]. We did not use their questionnaire because it was not adapted to the present study’s cultural context and was rather long (83 items).

Additionally, using the PSS-14 questionnaire in this study may have ‘masked’ the theoretically expected interaction effects between exposure to stressors and the factors protective of mental health. For example, a student with a high level of self-efficacy may not have realised that they had been exposed to a level of academic stressors that could have affected their mental health. Despite this limitation, the fact remains that the three potential health resources we measured were positively associated with perceived mental health. These resources can thus be considered promising starting points, both from a pedagogical and a clinical point of view, to work on improving these students’ mental health. To address this limitation, future studies should try, when possible, to identify the stressors to which their population is exposed. This could be done with less difficulties when population are more clearly defined than ours was, such as students from one institution or study domain.

Another limitation is the study’s cross-sectional design. This makes definitively labelling any causality between the different variables very difficult. Longitudinal studies might help in assessing causal relations between stress (or stressors if possible), protective factors of health, and mental health. Finally, the questionnaire was sent out in February 2020, just before the first wave of the COVID-19 pandemic struck Switzerland and health restrictions were imposed on many sections of society, notably students in tertiary education. It is possible that the associations we observed evolved during the crisis. It is thus important that future research continues to monitor mental health and its protective factors.

## Conclusion

The present study demonstrated that feelings of self-efficacy, a capacity for mindfulness, and the perception of social support were associated with the self-reported perceived mental health of first- and second-year bachelor’s degree students at a university of applied sciences in Switzerland. These potential factors protective of mental health provide three avenues of investigation for designing interventions to help students successfully navigate their academic life. They are particularly important because they have the potential to counteract or reduce the effects of exposure to unavoidable stressors. Furthermore, these factors are precious resources for protecting mental health during any life events where individuals might have to confront stressors, notably the transition from one’s studies to professional life, changes to family structures (e.g. divorce or death) or changes to one’s way of life such as the constraints imposed by the COVID-19 pandemic.

Thus, reinforcing a student’s personal toolbox of resources to better cope with academic life could not only help them to successfully complete their education and training—while preserving their mental health—but also endow them with useful skills for coping with stressful situations in their future professional life without suffering any psychological damage. Teaching students about these factors protective of mental health should become an essential component in the curricula of tertiary educational institutions in Switzerland.

## Data Availability

The data will be made available on SWISSUbase (www.swissubase.ch) when the larger study they form a part of is over. Meanwhile, they are available from the corresponding author upon reasonable request.

## Abbreviations

HES-SO: University of Applied Sciences and Arts Western Switzerland
HES: University of Applied Sciences
WHOQOL BREF: World Health Organisation Quality of Life Brief scale
PSS: Perceived Stress Scale
GSES: General Self-Efficacy Scale
MAAS: Mindful Attention Awareness Scale
MSPSS: Multidimensional Scale of Perceived Social Support
CER-VD: Human Research Ethics Committee of the Canton of Vaud
HPFS: Health Promotion Foundation of Switzerland
MBSR: Mindfulness-Based Stress Reduction
MBCT: Mindful-Based Cognitive Therapy
ACT: Acceptance and Commitment Therapy
DBT: Dialectical Behaviour Therapy
